# The effect of plant identity and the level of plant decay on molecular gut content analysis in a herbivorous soil insect

**DOI:** 10.1111/1755-0998.12032

**Published:** 2012-11-20

**Authors:** Corinna Wallinger, Karin Staudacher, Nikolaus Schallhart, Eva Peter, Philipp Dresch, Anita Juen, Michael Traugott

**Affiliations:** Mountain Agriculture Research Unit, Institute of Ecology, University of InnsbruckTechnikerstraße 25, 6020, Innsbruck, Austria

**Keywords:** *Agriotes*, click beetle, feeding experiment, *trn*L, wireworm

## Abstract

Plant roots represent an important food source for soil-dwelling animals, but tracking herbivore food choices below-ground is difficult. Here, we present an optimized PCR assay for the detection of plant DNA in the guts of invertebrates, using general plant primers targeting the *trn*T-F chloroplast DNA region. Based on this assay, we assessed the influence of plant identity on the detectability of ingested plant DNA in *Agriotes* click beetle larvae. Six different plant species were fed to the insects, comprising a grass, a legume and four nonlegume forbs. Moreover, we examined whether it is possible to amplify DNA of decaying plants and if DNA of decayed plant food is detectable in the guts of the larvae. DNA of the ingested roots could be detected in the guts of the larvae for up to 72-h post-feeding, the maximum digestion time tested. When fed with living plants, DNA detection rates differed significantly between the plant species. This may be ascribed to differences in the amount of plant tissue consumed, root palatability, root morphology and/or secondary plant components. These findings indicate that plant identity can affect post-feeding DNA detection success, which needs to be considered for the interpretation of molecularly derived feeding rates on plants. Amplification of plant DNA from decaying plants was possible as long as any tissue could be retrieved from the soil. The consumption of decaying plant tissue could also be verified by our assay, but the insects seemed to prefer fresh roots over decaying plant material.

## Introduction

Below-ground parts of plants such as roots and tubers represent an important food source for soil-dwelling animals (Zvereva & Kozlov 2012). But, our knowledge on below-ground herbivory still remains rudimentary (Hunter 2001; Johnson & Murray 2008). This can be mainly ascribed to the methodological hurdles linked to an examination of root feeding in the soil: the opaque habitat, the density of the root-felt, the inability to discriminate between the roots of different plant species and the minuteness of many of the contributing species make it hard to track what herbivores consume below the soil surface. Moreover, specific identification of semi-digested plant tissue in the intestine of herbivores is challenging. This is especially true for soil-living arthropods that consume their food in liquid stage. The identification of DNA from ingested food via molecular means is an elegant solution to reveal trophic relationships under natural conditions (King *et al*. 2008; Pompanon *et al*. 2012). However, the comprehensive body of literature concerning the identification of animal prey contrasts sharply with a small amount of studies focussing on their herbivorous counterparts. Only recently have PCR-based methods been successfully applied for the identification of ingested plants (Pegard *et al*. 2009; Valentini *et al*. 2009), including whole-body extracts of insects (e.g. Jurado-Rivera *et al*. 2009; Pumarino *et al*. 2011; Staudacher *et al*. 2011a). Considering the relatively short time that this approach has been used, the development of standardized and robust methodologies, that are broadly applicable, is still in progress. In this regard, the PCR assays described in Wallinger *et al*. (2012) represent a cost- and time-effective tool to screen large numbers of individual samples. These assays could also help to shed light into so far unstudied questions, such as the effect of identity on the detectability of DNA from ingested plants. So far, only two studies report on feeding experiments with herbivorous insects where DNA detection success was examined over time (Pumarino *et al*. 2011; Staudacher *et al*. 2011a). These studies indicate that differences in detection success of different food plants may occur but – to date – no one has explicitly investigated the effect of plant identity on DNA detectability in the gut contents of herbivores.

The importance of decaying plant material as a food source for soil-dwelling herbivores is another untouched issue. Apart from fresh plant tissue, soil-living arthropods might also commonly consume decaying plant material (Langenbuch 1932; Schaerffenberg 1942; Hemerik & de Fluiter 1999; Traugott & Juen 2007; Traugott *et al*. 2008). For example, plants that have been ploughed into the soil could be an important food source for soil-dwelling insect herbivores in arable land prior to the presence of the crop – a situation where the decaying plants represent the only potential food source at the herbivores' disposal. It is known from carnivorous arthropods that carrion prey is consumed regularly and that it can be detected by PCR as efficiently as fresh prey, irrespective of carrion age (Foltan *et al*. 2005; Juen & Traugott 2005). But, to our knowledge, it is currently unknown whether DNA of degraded plant food can be detected in the guts of soil-dwelling herbivores.

We addressed these questions in a series of experiments, in which we offered six different plant species, comprising a grass, a legume and four nonlegume forbs, to a soil-dwelling insect herbivore. Then, an optimized PCR assay for the detection of ingested plant DNA was employed to assess the influence of plant identity on its detectability during the course of digestion. Moreover, we tested whether plant DNA can be amplified from decaying plant material. Finally, we examined whether it is possible to detect DNA of decayed plant food in the guts of the herbivore. We chose larvae of click beetles (Coleoptera: Elateridae) of the genus *Agriotes* as our model herbivores. These so-called wireworms undergo a multiannual development in the soil (Parker & Howard 2001) and are widespread in arable land in Europe and North America (Staudacher *et al*. 2011b; Benefer *et al*. 2012). They are known to be generalists, feeding on the underground parts of a wide range of plants (Traugott *et al*. 2008) including a variety of crops, which makes them important pests (Hill 1987).

## Materials and methods

### Species and experimental setup

To gain a sufficient number of wireworms for the feeding experiments, we collected sixth- to eighth-instar larvae of *Agriotes* spp. (Eschscholtz 1829) in arable land in Mecklenburg-Vorpommern (Germany), around Wageningen (Netherlands) and in the Inn Valley (Tyrol, Austria). The larvae were kept separately in soil-filled transparent plastic tubes (20 mL) at 16 °C constant in the climatic chamber. Soil moisture in the tubes was visually checked every 2–3 days and the soil moistened if necessary. The wireworms were maintained on a diet of germinating wheat.

We examined plant DNA detection in wireworms either fed with decayed or fresh plants (hereinafter referred to as ‘feeding experiment’) and in decaying plant material (hereinafter referred to as the ‘plant decay experiment’). Because it is difficult to identify the larvae by morphological characters, this was done using a molecular identification key after the experiments (Staudacher *et al*. 2011c).

#### Feeding experiment with living grassland plants

Prior to the experiment seeds of ryegrass (*Lolium perenne L*.), red clover (*Trifolium pratense L*.), common yarrow (*Achillea millefolium L*.) and plantain (*Plantago lanceolata L*.) were individually sown in ten pots (15 × 15 × 20 cm) per plant species. Small plantlets of dandelion (*Taraxacum officinale* *Weber* *s*.*l.)* and burnet (*Pimpinella major (L.)* *Huds.)* were field collected and transferred into ten pots each. All pots were kept in a climatic chamber at 17 °C during daytime and 13 °C at night, which corresponds well to average soil temperatures in ∼5 cm depth in grasslands in the Inn Valley during the vegetation period. After 17 days, five wireworms, starved for 2 weeks prior to the experiment, were put into each pot, and they were then allowed to feed on the plants for 24 h. To estimate the amount of consumed plant material, the mass per larva of a subsample of 150 individuals was determined to the nearest 0.01 mg before and after feeding. Thereafter, the larvae were transferred to fresh soil-filled tubes and individually kept without food at 16 °C constant. Batches of 10–11 larvae were frozen at −28 °C at 0, 24, 48 and 72-h post-feeding, with individual wireworms stored in separate tubes.

#### Plant decay experiment

Maize (*Zea mays L*.) and wheat (*Triticum aestivum L*.) were chosen for the plant decay experiment. In October 2008, twenty maize stalks, together with soil from the field site, were transferred from an arable field (574 m a.s.l., Völs, Tyrol, Austria) to a climatic chamber. The plant material was buried at 25 cm depth in two separate soil-filled plastic containers (100 × 50 × 50 cm), kept at 15 °C constant and watered regularly. Likewise, over 100 four-week-old wheat plants (15–25 cm tall) were cut and buried in layers at 25 cm depth in two additional soil-filled plastic containers. Data loggers (Cyclobios, Innsbruck, Austria) were installed in each container to record soil temperature that ranged between 15 °C and 18 °C.

From November 2008 onwards, 10 of the twenty buried maize stalks were drawn randomly each month, and four tissue samples were taken per plant: from the cortex, the pith and the major as well as the fine roots. The remaining maize stalks were then put back in the containers until the next excavation. For the wheat, 10 plants were excavated weekly. Again, four samples were taken per plant: from the youngest leave, the primary foliage leaf, the stem and the roots. All samples were stored at −28 °C until DNA extraction, which was performed within the following weeks.

#### Feeding experiment with decayed plant material

This experiment comprised two different levels of decay of the food plants: plants decaying either for 1 week (decay 1) or 2 weeks (decay 2). Five-day-old plantlets of wheat (*T. aestivum*) were freeze-killed (−28 °C; for 2 weeks) and then allowed to individually decay in soil-filled plastic tubes (20 mL) in the climate chamber at 16 °C constant for one and 2 weeks, respectively. Fresh plants (control) served as control food to compare with the decayed food detection rates. One wireworm, which had been starved for at least 2 weeks, was added per tube where plantlets were buried near the surface. After a 24-h feeding period, each larva was transferred to a new soil-filled tube and kept without food at 16 °C constant. For each decay treatment, 10–11 larvae each were individually frozen at −28 °C at 0, 24, 48 and 72-h post-feeding.

### DNA extraction of wireworms and plants

For the feeding experiments, all reaction tubes containing frozen wireworms were checked for regurgitates; if present, the wireworms were transferred to fresh tubes, so that regurgitates and wireworms could be DNA extracted and tested separately.

To avoid amplification of DNA, which stems from plant material sticking on the outer body surface of the wireworms, we developed a method that successfully removes external plant DNA contaminations while not destroying the ingested DNA. This method is similar to previously published ones (Remén *et al*. 2010; Greenstone *et al*. 2011). To establish this method, 22 wheat-fed wireworms were artificially contaminated with maize by allowing them to crawl in chopped maize roots for 1 min. Then, they were freeze-killed at −80 °C. We knew from previous experiments that the maize DNA can be detected via PCR from such contaminated wireworms. We then carefully bathed 11 of the larvae in 1 mL of 1–1.5% sodium hypochlorite (‘bleach’; Sigma-Aldrich, St. Louis, Missouri, USA) for 30 s. The remaining 11 wireworms were washed with molecular-grade water only. Subsequently, all larvae were rinsed twice with molecular-grade water. Of the 11 ‘bleached’ wireworms, all tested negative for maize DNA (contamination of outer body surface) and all but two were positive for wheat (gut content). Half of the ones washed in water tested positive for both maize and wheat and half for wheat only. In previous experiments, bleaching for varying time spans ranging between 30 s and 12 min were tested: we found that keeping the larvae for 30 s in bleach was sufficient to remove DNA contamination of the outer body surface. Based on these findings, we established the following wash protocol for wireworms: first, the wireworms were cleaned of any plant material potentially sticking on their outer body surface prior to extraction. Second, each larva was carefully washed in 1 mL of 1–1.5% sodium hypochlorite solution (Sigma-Aldrich) for 30 s. Subsequently, the larva was rinsed twice with molecular-grade water. Then, the head capsule and the 9th abdominal segment of each wireworm were cut off, and the remaining middle part of the larva, including its intestinal tract, was homogenized with 3-mm glass beads for 1 min at 5000 rpm using a Precellys® 24 Tissue Homogenizer (Bertin Technologies, Montigny-le-Bretonneux, France). For DNA extraction, a CTAB-based protocol described in Juen and Traugott (2005) was employed with the following modifications to increase the DNA yield: the extraction buffer contained 430-μL TES buffer (final concentrations 0.1 m TRIS, 10 mm EDTA, 2% SDS; pH 8), 10-μL Proteinase K (20 mg/mL) and 10-mg Polyvinylpyrrolidone (PVP), and samples were incubated overnight at 58 °C.

As regurgitates are a much smaller sample than whole wireworms and because they are likely to contain mainly food DNA and to a much lesser extent DNA of the consumer (Waldner & Traugott 2012), each regurgitate was immediately dissolved in extraction buffer and incubated for 2 h only. All extractions were done in a separate pre-PCR laboratory using a UVC-equipped laminar flow hood, and two extraction-negative controls were included in each batch of 30 samples to check for cross-sample contamination. For further analysis, the PCR results of wireworms and their corresponding regurgitates were combined, that is, individuals were counted positive if at least one of the samples (larva or regurgitate) was positive for a specific plant taxon.

Wheat and maize plants of the plant decay experiment were DNA extracted, following a CTAB-based protocol described in Wallinger *et al*. (2012).

### PCR and electrophoresis

For the detection of plant DNA in the feeding experiments with the living plant species, a new PCR assay was established. It employs general plant primers accessing the *trn*L chloroplast DNA region: 5′-CGA AAT CGG TAGA CGC TAC G-3′ (primer c A49325*,* situated in the *trn*L (UAA) exon; Taberlet *et al*. 1991) and 5′-GAT TTG GCT CAG GAT TGC CC-3′ (*trn*L110R, located in the *trn*L (UAA) intron; Borsch *et al*. 2003). PCR amplifications were performed in 15-μL reaction mixtures containing 4-μL DNA extract, 7.5-μL 2× Type-it Mutation™ Detect PCR Kit (Qiagen, Hilden, Germany), 0.5-μL 5× Q-Solution (Qiagen), 0.5-μg BSA and 0.5 μm of each general plant primer. The thermocycling programme was as follows: 95 °C for 5 min, 35 cycles of 92 °C for 20 s, 60 °C for 30 s and 70 °C for 30 s and a final elongation of 70 °C for 5 min.

To measure the sensitivity of this newly established PCR assay, DNA templates of 13 selected plant species (Table 1) were generated according to the procedure described in Sint *et al*. (2012). General plant primers accessing the *trn*T-F cpDNA region (Taberlet *et al*. 1991) were used to amplify fragments, which covered the binding sites of the currently used primers. Based on the DNA concentrations of the purified PCR products, determined with a VICTOR™ *×*4 Multilabel Plate Reader (Perkin Elmer, Waltham, Massachusetts, USA) and the Quant-iT™ PicoGreen® dsDNA Assay Kit (Invitrogen, Paisley, UK), the number of template copies per μL DNA extract was calculated as described in Sint *et al*. (2012), which was finally used for sensitivity testing. The actual sensitivity of the optimized diagnostic PCR assay was determined via serial dilution of template DNA, that is, known numbers of single-stranded copies; see Sint *et al*. (2012) for further details. We tested standardized DNA templates containing 100 000; 20 000; 10 000; 2000; 400; 200; 100; 50; 25 single-stranded DNA template molecules per μL. As we used 4 μL of the standardized DNA templates in our PCR assay, 100 template molecules were present in the PCR at the lowest concentration tested.

**Table 1:** Plant species list used for determining sensitivity of the general plant primers c A49325 (Taberlet *et al*. 1991) and *trn*L110R (Borsch *et al*. 2003). The lowest template numbers (copies per reaction) where a PCR product could be obtained are given as detection limits

			Detection limits of plant DNA	
			
Plant species	Plant family	Functional group	Plant DNA only	Plus wireworm DNA
Achillea millefolium*	Asteraceae	Herb	100	100
*Fagopyrum esculentum*	Polygonaceae	Herb	100	100
Lolium perenne*	Poaceae	Grass	100	100
*Lupinus angustifolius*	Fabaceae	Legume	100	100
*Phaseolus coccineus*	Fabaceae	Legume	200	200
Pimpinella major*	Apiaceae	Herb	100	100
Plantago lanceolata*	Plantaginaceae	Herb	100	100
*Sinapis alba*	Brassicaceae	Herb	100	100
Taraxacum officinale*	Asteraceae	Herb	100	100
Trifolium pratense*	Fabaceae	Legume	100	100
*Trifolium repens*	Fabaceae	Legume	100	100
*Triticum aestivum*	Poaceae	Grass	100	100
*Zea mays*	Poaceae	Grass	100	100

* Plant species that were fed to wireworms in the feeding experiment with living grassland plants.

Assay sensitivity was also evaluated in the presence of wireworm DNA to test the capability of this PCR for molecular gut content analysis. For each plant species (Table 1), 1 μL of the two lowest concentrations of template DNA, which tested positive were spiked with 3.5 μL of undiluted *Agriotes* spp. DNA.

The DNA extracts of both the plant decay experiment and the feeding experiment with decayed plants were tested in a multiplex PCR assay specifically targeting short plastid DNA fragments of maize and wheat (TZ duplex, described in Wallinger *et al*. (2012)). The PCR conditions followed the description provided in Wallinger *et al*. (2012) for the TZ duplex: 15-μL reactions included 4-μL DNA extract, 7.5-μL 2× Type-it Mutation™ Detect PCR Kit (Qiagen), 0.5-μL 5× Q-Solution (Qiagen), 0.5-μg bovine serum albumin (BSA), 4-mm MgCl_2_ and the primers at a final concentration of 0.2 μm and 0.5 μm for maize and wheat, respectively. The cycling protocol included 5 min at 95 °C, 40 cycles of 20 s at 92 °C, 90 s at 54 °C, 90 s at 70 °C and a final elongation of 5 min at 70 °C.

One positive (DNA of *Z. mays*) and 3–5 negative controls (PCR-grade water instead of DNA) were run within each PCR assay to check for amplification success and DNA carry-over contamination, respectively.

All PCR products were visualized on QIAxcel, an automated capillary electrophoresis system (Qiagen) with method AL320, and the results were scored using BioCalculator Fast Analysis Software version 3.0 (Qiagen). All samples showing the expected fragment length and with a signal above 0.1 relative fluorescent units were deemed to be positive. The wireworm DNA extracts of the feeding experiments that tested negative in a first run were re-tested in a second PCR to increase the chances of amplification for samples, which contained only minute quantities of target DNA.

### Statistical analyses

The influence of digestion (=time post-feeding) on plant DNA detection success was tested in both the living and decaying plant feeding experiments. We employed a logit regression model as the dependent variables were binomially distributed. Overall plant DNA detection rates were tested for significant differences between levels of decay and plant species, respectively, using chi-squared tests. All calculations were done in PASW Statistics 18 (IBM, Armonk, New York, USA).

## Results

### Feeding experiments with living grassland plants

The new PCR assay proved to be highly sensitive for all but one plant species tested; amplification and visualization of the target DNA was possible down to the presence of 100 target template molecules per PCR; in *Phaseolus coccineus L*. the detection limit was 200 templates. The presence of wireworm DNA did not affect PCR sensitivity (Table 1).

In total, 264 *Agriotes* larvae were analysed in our feeding experiments: 46 fed with *T. pratense*, 45 fed with *T. officinale*, 44 fed with *L. perenne* and *P. lanceolata*, 43 fed with *A. millefolium* and 42 fed with *P. major*. The molecular identification of the wireworms assigned 111 individuals to *A. obscurus,* 75 to *A. lineatus/proximus,* 41 to *A. ustulatus* and 32 to *A. sputator* (five specimens could not be identified). The mean mass of wireworms at the beginning of the experiment was 24.9 mg (±12.6 SD). After the 24-h feeding period, the average change in mass (meal size) for fed larvae was 0.8 mg (±1.4 SD; ranging between -4.9 and 5.2 mg).

**Fig. 1:**
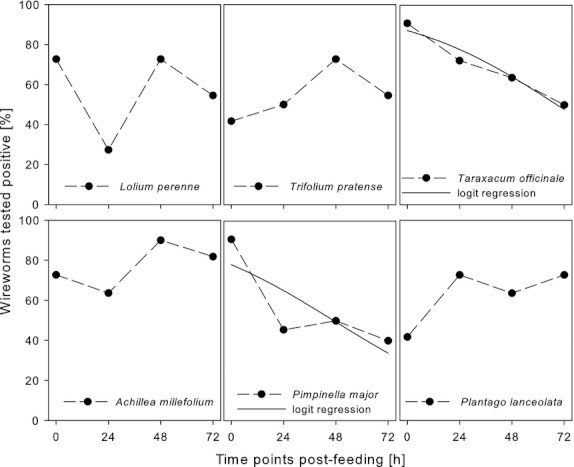
Detectability of plant DNA in the guts of *Agriotes* spp. larvae fed with six different plant species using general plant primers. A minimum of 10 larvae was tested at each time point post-feeding (0, 24, 48 and 72 h).

DNA of the ingested plants could be detected in the guts of wireworms for up to 72-h post-feeding, the maximum digestion time in our experiments (Fig. 1). The fragment length of this plant DNA varied between 120 bp and 123 bp. Detection rates were high across the board, considering all time points. Wireworms fed with *A. millefolium* exhibited maximum overall detection rates with 76.7%. For those fed with *P. lanceolata* 70.5% tested positive*,* followed by 68.9% fed with *T. officinale,* 57.1% fed with *P. major*, 56.8% fed with *L. perenne* and 54.3% fed with *T. pratense*. The detection rates for ingested plant DNA differed significantly between *A. millefolium* and three plant species: *L. perenne* (χ^2^ = 3.886, *P* < 0.05), *P. major* (χ^2^ = 6.746, *P* < 0.01) and *T. pratense* (χ^2^ = 4.911, *P* < 0.05). No such difference occurred between *A. millefolium* and *P. lanceolata* or *T. officinale*.

The detectability of plant DNA decreased significantly with time in wireworms fed with *T. officinale* (*P* = 0.039, *R*^2^ = 0.102 [Cox & Snell], *R*^2^ = 0.143 [Nagelkerkes]) and in larvae, which consumed *P. major* (*P* = 0.034, *R*^2^ = 0.112 [Cox & Snell], *R*^2^ = 0.15 [Nagelkerkes]) (Fig. 1), while detection success was independent of digestion time for the rest of the plant species. Plant detection success was independent of the wireworm species.

### Plant decay experiment

The buried wheat plants decomposed much faster than the maize stalks. The primary foliage leaves of wheat were totally decomposed 2 weeks after burying, whereas the youngest wheat leaves remained detectable for up to 37 days. The sampling of wheat ended 67 days after the start of the experiment because the tissue of the buried wheat plants was completely decomposed later on. In maize, parts of the ligneous maize stalks remained detectable in the containers until 329 days after the start of the experiment. Only the fine maize roots were totally decomposed after 91 days (except for one individual, which was sampled 121-day post-burying).

DNA extraction started with those samples that decayed for the longest time, including three samples per plant part. All of them tested positive with the TZ duplex, which included the specific primers for wheat and maize (detection rate = 100%), indicating that DNA is detectable as long as plant tissue could be retrieved from the soil. Hence, we refrained from analysing any of the samples taken at earlier time points.

### Feeding experiments with decayed plant material

In this experiment, a total of 160 *Agriotes* larvae were analysed: 75 for control*,* 42 for decay 1 and 43 for decay 2. The molecular species identification revealed 57 *A. sputator,* 42 *A. ustulatus,* 30 *A. lineatus/proximus,* 29 *A. obscurus* and 1 *A. sordidus* (one individual could not be molecularly identified).

**Fig. 2:**
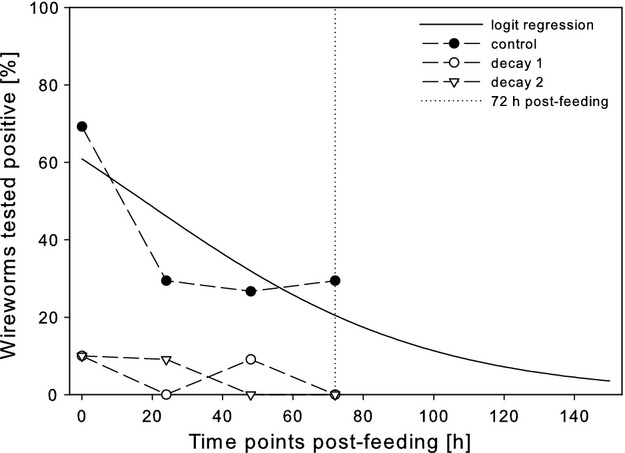
Detectability of plant DNA in the guts of *Agriotes* spp. larvae fed with wheat plants that were allowed to decay for 1 week (decay 1) and 2 weeks (decay 2). The control group was fed with fresh wheat. A minimum of 10 larvae was tested at each time point post-feeding (0, 24, 48 and 72 h).

The DNA of the decayed plant food could be amplified from the gut content of the wireworms for up to 72-h post-feeding, the maximum digestion time in our experiments (Fig. 2). While in almost half of the wireworms fed with fresh wheat plants DNA could be detected (overall detection rate = 42.7%), this was true for only 4.8% and 4.7% for the decay 1 and 2, respectively. The overall detection rates differed significantly between control and decay 1 (χ^2^ = 18.764, *P* < 0.001) and control and decay 2 (χ^2^ = 19.256, *P* < 0.001). No such difference occurred between the two decay levels. In wireworms fed with fresh wheat, the detectability of plant DNA decreased significantly with time (*P* = 0.007, *R*^2^ = 0.101 [Cox & Snell], *R*^2^ = 0.135 [Nagelkerkes]), while the generally low detection success of the two decay levels was independent of the length of digestion time (Fig. 2). There was no correlation between wireworm species and plant DNA detection success.

## Discussion

The present experiments showed that both identity and level of decay of plant food affect the DNA detection success within the gut contents of wireworms. Moreover, our findings indicate that amplifiable DNA is present in decaying plants as long as plant material can be retrieved from the soil. These results also proved the high sensitivity of the newly established PCR assay.

When feeding the *Agriotes* larvae with fresh plants, all six plant species, comprising legumes, grasses and nonlegume forbs, were detectable for the maximum digestion time tested. Similar to the experiment described in Staudacher *et al*. (2011a), the initial mass of the wireworms and their meal size did not affect plant DNA detection rates (data not shown). Still, specific differences occurred between the different plants, which may be ascribed to differences in the amount of root material consumed, or to their palatability due to varying root morphology and secondary plant components. In the present experiment, plants exhibiting different root forms were included: *L. perenne,* a subcosmopolitan in temperate grasslands (Peeters *et al*. 2004), can build up a dense root-felt within the upper soil layer (Kutschera & Lichtenegger 1982), while *A*. *millefolium* forms extensive rhizomes with numerous rootlets (Hänsel *et al*. 1992; Kutschera & Lichtenegger 1992). The other herb species tested within the current study form taproots (Kutschera & Lichtenegger 1982). Gut content DNA detection rates differed significantly between these different root forms, exhibiting significant higher detection rates for rhizomatous plants (i.e. *A*. *millefolium*) compared with the root-felt-forming (*L. perenne*) and to taproot-forming (*P. major* and *T. pratense*) species. No differences occurred when comparing *A*. *millefolium* with the rosette-growing species *T. officinale* and *P. lanceolata*. Likewise, Miles & Petherbridge (1927) found wireworms in high numbers at the roots of *A. millefolium*, while very few larvae occurred near roots of chickweed (*Stellaria media (L.) VILL. Agg.)*, goutweed (*Chenopodium album L.)* and annual nettle (*Urtica urens L*.).

Assumed that the observed differences in plant DNA detection rates are based on different feeding rates (i.e. high feeding rates correlate with high detection rates), the current results are indicating that wireworms prefer the rhizome- (*A*. *millefolium*) and rosette-forming plants (*T. officinale* and *P. lanceolata*) to those with tap roots and the fine-rooted plant species. This could then be explained by optimal foraging (Mac Arthur & Pianka 1966), because handling times are potentially higher for plants with fine roots than for thick rhizomes. In mesocosm experiments Schallhart *et al*. (2012) *Agriotes* wireworms also exhibited preferences for specific plants. The two most preferred plant species within the present experiment, *A*. *millefolium* and *T. officinale,* belong to the Asteraceae. Most of the secondary compounds in this family have a low effect on insects (Frohne & Jensen 1992). The authors ascribe this to the fact that the Asteraceae are an evolutionarily relatively young plant family that rarely hosts monophagous insects.

In the feeding experiment with decayed plant material, plant DNA detection rates were low compared with the experiment with living plant food. These results indicate a low affinity of the wireworms to decaying plants. This is in accordance with observations on soil-dwelling carnivorous carabid larvae, where prey consumption was negatively correlated with cadaver age (Juen & Traugott 2005). However, unlike carnivorous arthropods, whose food intake within one meal can reach 50% of their initial biomass, *Agriotes* wireworms show much smaller meal sizes, averaging 3% when feeding them with maize and wheat (Staudacher *et al*. 2011a). Interestingly, wireworms where no gain in weight or even a biomass loss was observed after feeding also tested positive for plant DNA. Although it is not straightforward to determine whether a larva had been feeding and how much root material it was consuming, the present results indicate that the food DNA was detectable, independent of the plants' level of decay or the digestion period. Likewise, the detection success of carrion prey in the guts of carabid beetles was independent of their level of decay (Foltan *et al*. 2005; Juen & Traugott 2005).

DNA was amplifiable from decaying plant material as long as any tissue could be retrieved from the soil, which also means that plant DNA from soil samples or the gut contents of primary decomposers will be detected by PCR. This would offer, for example, new avenues to examine how the litter from different plants is utilized by different decomposers such as earthworms, complementing other approaches such as isotopic labeling (e.g. Seeber *et al*. 2010). Care needs to be taken, however, in those studies where it is important to differentiate between living and dead plant material as, for example, the assessment of below-ground plant species richness (e.g. Hiiesalu *et al*. 2012) or when dead plant material from the soil can potentially contaminate samples.

Besides examining the impact of identity and the level of decay of plant food on gut content DNA detection, we aimed to provide a standardized and robust methodology to test herbivorous insects for the presence of plant DNA in their guts. Detection success of ingested plant DNA was similar to PCR assays including two different plant genes as general markers (*trn*L and *rbc*L) and specific primers (Staudacher *et al*. 2011a). Taberlet *et al*. (2007) promoted a primer combination targeting the p6 loop for ecological applications, such as the identification of degraded DNA via sequencing (Jurado-Rivera *et al*. 2009; Valentini *et al*. 2009; Navarro *et al*. 2010; Schnell *et al*. 2010). But, the short amplicon (∼90 bp) is suboptimal for diagnostic PCR because it is difficult to differentiate the bands from primer dimers. In combining the forward primer *c* A49325 (Taberlet *et al*. 1991) with the reverse primer *trn*L110R (Borsch *et al*. 2003), we gained both, high sensitivity and high efficiency in detecting degraded plant DNA. The size of the amplicon of ∼120 bp is small enough to detect degraded DNA and large enough to provide bands also detectable on standard agarose gel electrophoresis. It works on a large variety of plants from different families (137 species, 24 families, data not presented). Due to its larger size, the current amplicon also bears additional sequence information for species identification via barcoding. The comparatively simple PCR-based assay presented here could be highly useful for studies examining the diet of herbivores, providing a means to exclude samples, which contain no plant DNA from further analysis (i.e. species-specific plant identification), thus reducing time and cost requirements. The current PCR assay also represents an ideal combination with next generation sequencing, a method that has been gaining importance in trophic ecology (Glenn 2011; Pompanon *et al*. 2012). Implementing the present PCR assay as a first step, the sequencing efforts will be restricted to those samples that contain plant DNA.

The current study represents a first experimental comparison of DNA detection rates for different plant species as well as decayed plant tissue in the gut contents of insect herbivores via diagnostic PCR. The observed differences in detection rates between different plant species represent a qualitative assessment of the dietary choice only. But, although we could not determine the amount of plant tissue per plant species consumed, the results indicate that plant identity affects the dietary choice of *Agriotes* larvae. Moreover, these wireworms seem to depreciate decayed plants as food source. This is supported by earlier work on *Agriotes ustulatus* where successful development was observed only when fresh plant material was available (Furlan 1998). Similarly, in *Agriotes obscurus,* no significant consumption of soil organic matter was found (Traugott *et al*. 2007, 2008). An alternative explanation would be that DNA of decaying plant food is less detectable in the guts, a hypothesis which needs to be tested in future experiments.

## Notes

C.W. was responsible for the conception and design of the study, optimizing and performing the PCR assays, evaluating the primer sensitivity, drafting the manuscript, and compiling the final version to be published. K.S. performed the experiment with decayed plants, did the DNA extraction, the measurement of DNA concentrations, contributed to the optimization and performance of PCRs and the evaluation of primer sensitivity, and critically revised the article. N.S. was responsible for the design of the study, supervised the decay experiments and critically revised the article. E.P. and P.D. performed the decay- and the feeding experiment with fresh plants, respectively and did the DNA extraction of these samples. A.J. designed the plant primers and M.T. was responsible for the conception and design of the study, critically revising the article and the final approval of the version to be published.

## Data accessibility

Raw data of feeding experiments with fresh and decaying plants: DRYAD entry: doi:10.5061/dryad.p7c8s.
